# Expression of Cystathionine β-synthase and Cystathionine γ-lyase in Human Pregnant Myometrium and Their Roles in the Control of Uterine Contractility

**DOI:** 10.1371/journal.pone.0023788

**Published:** 2011-08-23

**Authors:** Xing-Ji You, Chen Xu, Jian-Qiang Lu, Xiao-Yan Zhu, Lu Gao, Xiao-Rui Cui, Yuan Li, Hang Gu, Xin Ni

**Affiliations:** 1 Department of Physiology, Second Military Medical University, Shanghai, China; 2 Department of Obstetrics and Gynecology, Changhai Hospital, Second Military Medical University, Shanghai, China; 3 Department of Biomechanics, Shanghai University of Sports, Shanghai, China; Florida International University, United States of America

## Abstract

**Background:**

Human uterus undergoes distinct molecular and functional changes during pregnancy and parturition. Hydrogen sulfide (H_2_S) has recently been shown to play a key role in the control of smooth muscle tension. The role of endogenous H_2_S produced locally in the control of uterine contractility during labour is unknown.

**Methodology/Principal Findings:**

Human myometrium biopsies were obtained from pregnant women undergoing cesarean section at term. Immunohistochemistry analysis showed that cystathionine-γ-lyase (CSE) and cystathionine-β-synthetase (CBS), the principle enzymes responsible for H_2_S generation, were mainly localized to smooth muscle cells of human pregnant myometrium. The mRNA and protein expression of CBS as well as H_2_S production rate were down-regulated in labouring tissues compared to nonlabouring tissues. Cumulative administration of L-cysteine (10^−7^–10^−2^ mol/L), a precursor of H_2_S, caused a dose-dependent decrease in the amplitude of spontaneous contractions in nonlabouring and labouring myometrium strips. L-cysteine at high concentration (10^−3^ mol/L) increased the frequency of spontaneous contractions and induced tonic contraction. These effects of L-cysteine were blocked by the inhibitors of CBS and CSE. Pre-treatment of myometrium strips with glibenclamide, an inhibitor of ATP-sensitive potassium (K_ATP_) channels, abolished the inhibitory effect of L-cysteine on spontaneous contraction amplitude. The effects of L-cysteine on the amplitude of spontaneous contractions and baseline muscle tone were less potent in labouring tissues than that in nonlabouring strips.

**Conclusion/Significance:**

H_2_S generated by CSE and CBS locally exerts dual effects on the contractility of pregnant myometrium. Expression of H_2_S synthetic enzymes is down-regulated during labour, suggesting that H_2_S is one of the factors involved in the transition of pregnant uterus from quiescence to contractile state after onset of parturition.

## Introduction

Human myometrium undergoes profound structural and functional changes during pregnancy and labour. Throughout most of the gestation, the myometrium activity is characterized by poorly coordinated contractures. In late pregnancy, the uterus undergoes preparedness for the stimuli that lead to contractility and labour[1], [2]. The intracellular processes involved in this shift from smooth muscle relaxation to contraction at the time of parturition are largely unknown, however, it is suggested that this transformation results from the coordinated expression of a cassette of contraction-associated proteins such as, gap junction and the receptors of agonists (such as oxytocin and prostaglandins)[1], [3]–[4].

Hydrogen sulfide (H_2_S) has now been proposed to be the third endogenous gaseous transmitter besides nitric oxide and carbon monoxide [5], [6]. Endogenous H_2_S is generated from L-cysteine principally through the activity two pyridoxal-5-phosphate-dependent enzymes: cystathionine-γ-lyase (CSE, EC 4.4.1.1) and cystathionine-β-synthetase (CBS, EC 4.2.1.22) [6]–[8]. CBS and CSE are widely distributed in tissues, however, CBS activity is 30-fold greater than CSE in brain whereas CSE expression and activity are much higher than CBS in the cardiovascular system[9], [10]. Expression of CBS and CSE have also been found in smooth muscle tissues. CSE is the predominant enzyme responsible for H_2_S generation in vasculature smooth muscle[11], whereas both CBS and CSE contribute to H_2_S generation in gastrointestinal and penial smooth muscle[12], [13]. Recently, Patel et al[14] showed that CBS and CSE enzymes exist in rat and human nonpregnant and pregnant myometrium tissues, and rat uterus homogenate is capable of producing H_2_S from its precursor L-cysteine *in vitro*. However, the roles of endogenous H_2_S produced locally in uterus has not well been defined.

H_2_S modulation of smooth muscle tone has been demonstrated in various smooth muscle tissues. It seems that H_2_S has either relaxant or excitatory effect on contractile activity of smooth muscle. H_2_S has been shown to cause relaxation in vasculature, bronchial and intestinal smooth muscles [11], [15]–[18]. In another hand, it has also been shown to enhance the vascular tension [19] and provoke tachykinin-mediated bronchoconstriction [20]. Sidhu et al [21] recently reported that H_2_S inhibits spontaneous contractility of rat uterine strip *in vitro*. To date, there are no studies examining the roles of endogenous-produced H_2_S in regulating the contractility of human uterine smooth muscle in particular, during pregnancy and labour. Investigation of this issue would expand our knowledge and gain insight of the mechanisms controlling human parturition. Therefore, in the present study, we examined the localization of CSE and CBS in human pregnant myomtrium at first, and then determined the level of CBS and CSE as well as H_2_S production rate in pregnant myometrium taken from women undergoing labour or not undergoing labour. Finally, we investigated the effects of H_2_S generated locally through its synthetic enzymes on spontaneous contractility of human pregnant myometrium.

## Results

### Localization of CBS and CSE in human pregnant myometrium

**Figure 1:**
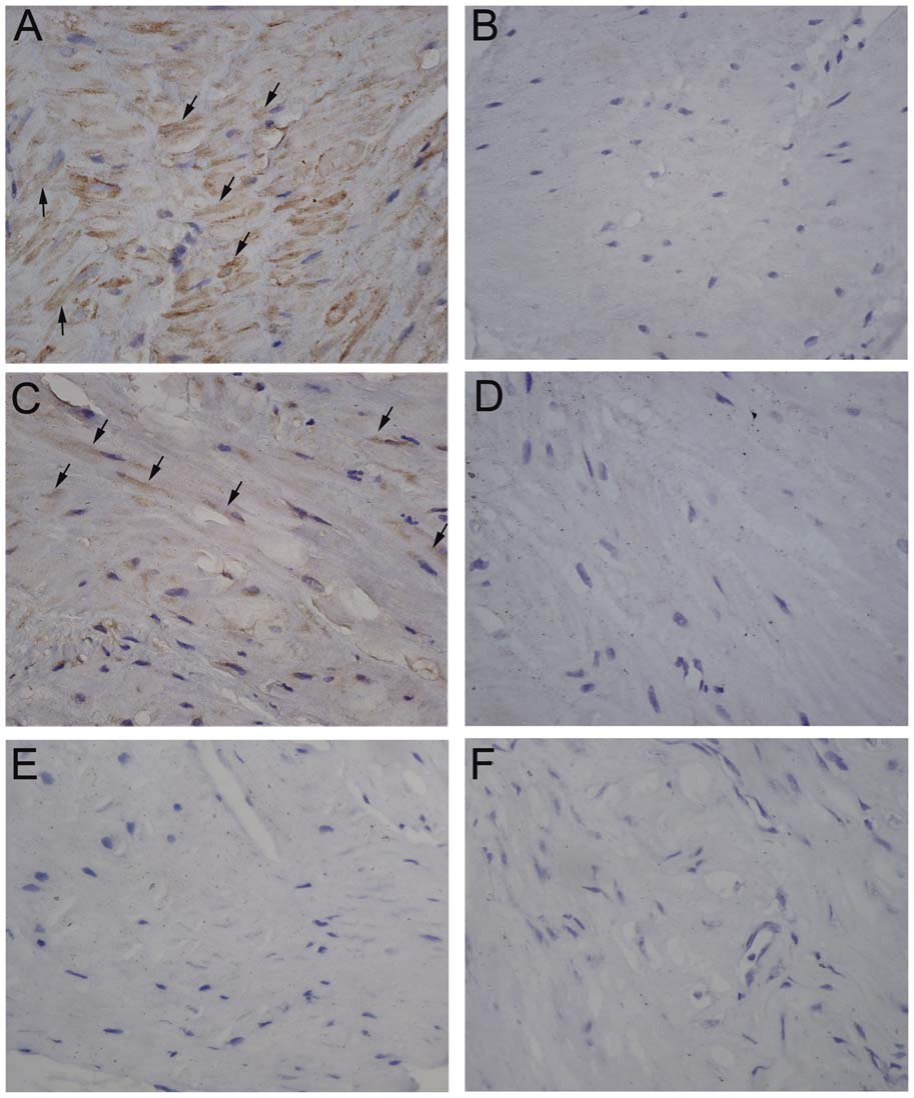
The expression of CBS and CSE in pregnant human myometrium. A&C shows representative sections for positive staining for CBS (A) and CSE (C). B&D shows the negative control sections stained with CBS preabsorption antibody (B) and CSE preabsorption antibody (D). E&F, negative control sections, the primary antibody was substituted by normal IgG(E) and PBS(F). Arrow: positive staining in myometrium smooth muscle cells. Original magnification ×400.

Immunohistochemistry shows that positive immunoreactivity for CBS as well as CSE was predominantly localized to smooth muscle cells of myometrium (Fig. 1A,C). The positive staining of these proteins was also found in smooth muscle lining blood vessel. Immunoreactivity was abolished when the antibody was preabsorbed with excess peptide, thereby confirming the specificity of the antibodies (Fig 1B &D).

### The expression of CBS and CSE as well as H_2_S production rate in pregnant myometrium before and during labour

**Figure 2:**
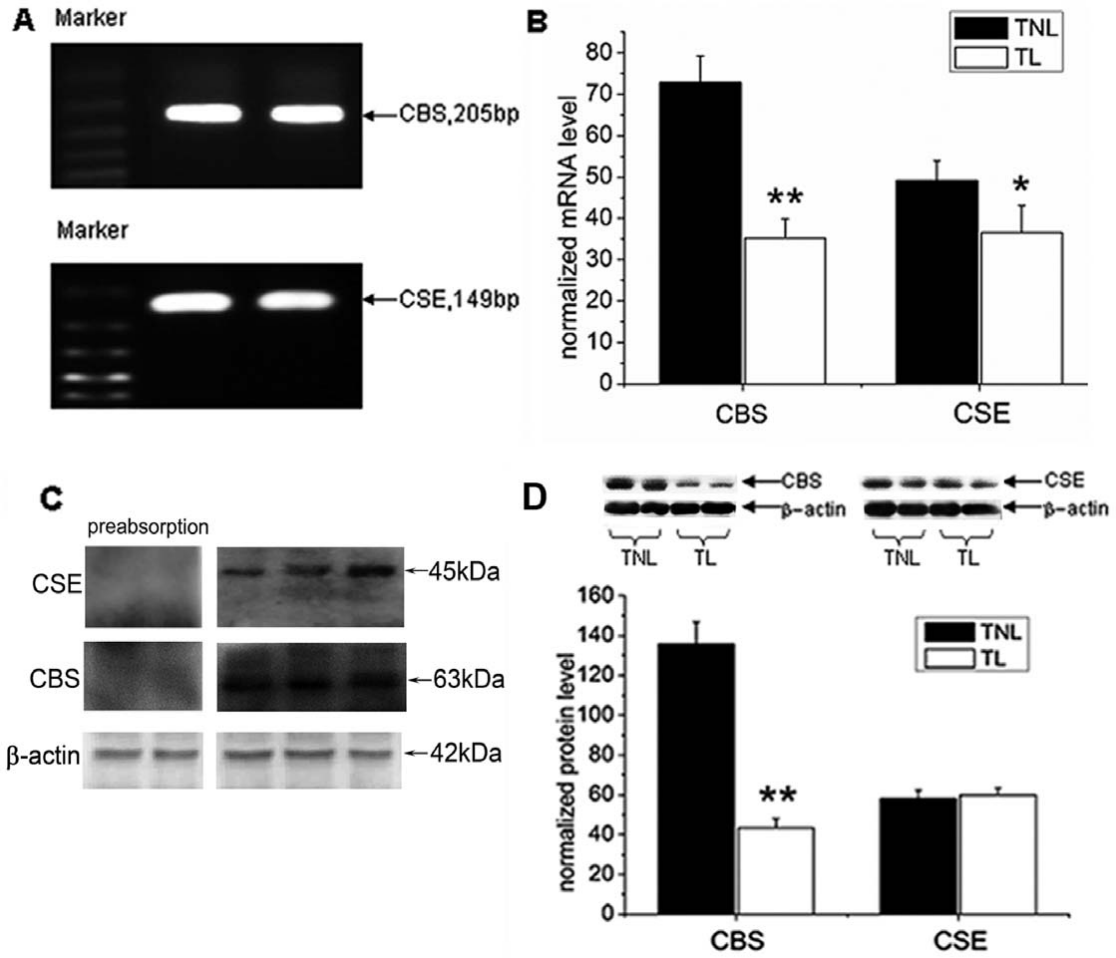
CBS and CSE expression in pregnant myometrium before and after onset of labour. Myometrial tissues were obtained from pregnant women at term before the onset of labour (TNL, n = 10) or during active labour (TL, n = 10) A, the RT-PCR products of CBS and CSE. B, cumulative data of CBS and CSE mRNA levels in TNL and TL myometrium samples. The mRNA levels of CBS and CSE were determined by quantitative real-time RT-PCR as described in Material and Methods. Data were presented as mean±SEM. *P<0.05, **P<0.01 vs TNL. C, Western blot analysis of CBS and CSE in human pregnant myometrium. D, semiquantitation of Western blot signals of CBS and CSE in TNL and TL myometrium biopsies. Representative protein bands for CBS and CSE were on the top of histogram. Data were presented as mean±SEM.. **P<0.01 vs TNL.

RT-PCR analysis detected both CBS and CSE mRNA expression in pregnant myometrium biopsies (Fig. 2A). It has been shown by real-time quantitative PCR that mRNA levels of CBS and CSE were down-regulated in TL samples compared with TNL samples (*P*<0.01 for CBS; *P*<0.05 for CSE; Fig.2B).

Western blot analysis was performed to determine protein expression of CBS and CSE. It recognized a band of approximately 63 kDa corresponding to CBS and a band of about 45 kDa corresponding to CSE. The level of CBS protein expression was significantly decreased in TL group compared with TNL group (*P*<0.01). However, no significant differences in CSE protein expression were found between TNL and TL groups (Fig.2D).

**Figure 3:**
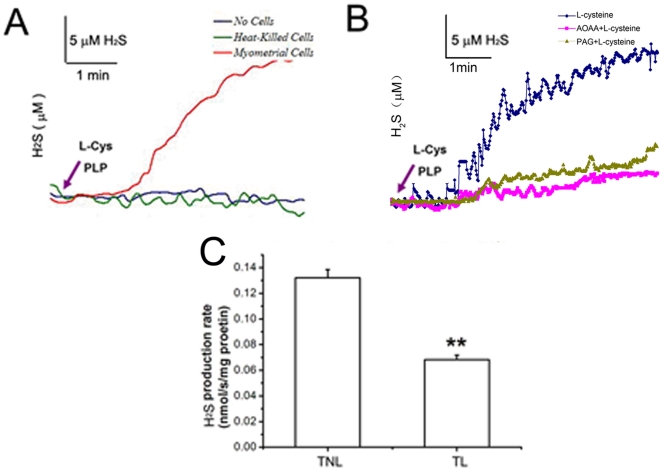
H_2_S production rate in human TNL and TL myometrium. A, Representative traces of H_2_S production in the homogenate of myometrium tissues. H_2_S production was initiated by the addition of L-cysteine and PLP. There were two control traces of buffered solutions without tissue or with heat-killed tissue containing L-cysteine and PLP, indicating no spontaneous H_2_S production. B, Representative traces of AOAA and PAG blocking H2S production by the addition of L-cysteine in the homogenate of myometrium tissues. C, cumulative data of H2S production rate in TNL (n = 13) and TL myometrium (n = 6). Data were presented as mean?SEM. **P<0.01 vs TNL. L-Cys: L-cysteine.

The homogenate of the pregnant myometrium tissue was able to produce H_2_S after addition of L-cysteine. This effect was blocked by either CSE inhibitor PAG or CBS inhibitor AOAA (Fig. 3A&B). The H_2_S production rate was significantly decreased in TL myometrium biopsies compared with TNL samples (*P*<0.01) (Fig. 3C).

### Effects of H_2_S on spontaneous contractility and baseline muscle tension of pregnant myometrium

Endogenous H_2_S is generated from L-cysteine. Thus, L-cysteine was used to explore the effects of endogenous H_2_S generated by CBS and CSE on the contractility of human myometrium *in vitro*.

**Figure 4:**
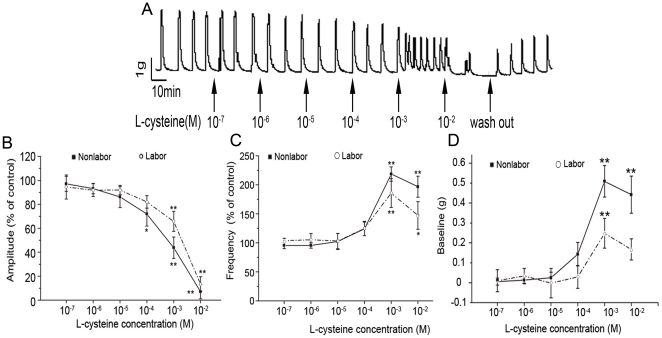
The effects of L-cysteine on spontaneous contractions and baseline muscle tension of myometrium. Human myometrium strips were obtained from TNL patients (n = 6) and TL patients (n = 4) undergoing cesarean section. Cumulative increases in L-cysteine (10^−7^–10^−2^ mol/L) were applied to myometrial strips. A, representative recording of contractions of nonlabouring myometrial strips. B,C&D, cumulative data of amplitude (B), frequency (C) and baseline tone (D). Data of amplitude and frequency were presented as mean (SEM) percentage of the results obtained before any drug application for each individual strip (control). For baseline muscle tone, the control was set as “0 ”. Data were presented as mean±SEM. *P<0.05, **P<0.01 vs control.

All of uterine strips exhibited spontaneous contractile activity *in vitro*. The strips were treated with a cumulative increase in concentrations of L-cysteine (10^−7^–10^−2^ mol/L). As shown in Fig 4, L-cysteine caused a dose-dependent decrease in amplitude of spontaneous contractions in both TNL and TL groups. L-cysteine showed more potent effect on contraction amplitude in TNL strips than in TL strips. The IC50 value of L-cysteine was 6.98×10^−4^±4.26×10^−4^ mol/L in TNL group whereas it was 2.24×10^−3^±1.1×10^−3^ mol/L in TL group. The difference in IC50 value between TNL and TL groups was significant (*P*<0.01). L-cysteine at 10^−3^ mol/L significantly enhanced the frequency of spontaneous contractions, with about 118% increase in TNL group and about 81% increase in TL group.

Application of L-cysteine induced tonic contraction in both TNL and TL strips at high concentration (10^−3^ mol/L). The tonic contraction was increased from 0 g (basal) to 0.51 ± 0.08g in TNL group and to 0.25 ± 0.07g in TL group.

**Figure 5:**
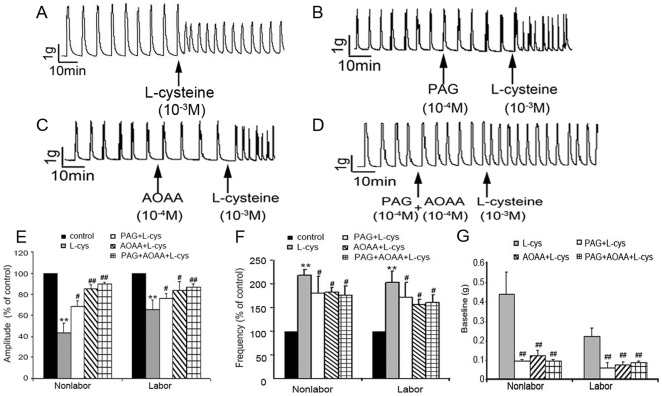
CSE and CBS inhibitor reverse the effects of L-cysteine on contractility of myometrium. CSE inhibitor PAG (10^−4^mol/L) and CBS inhibitor AOAA(10^−4^mol/L) was applied to myometrial strips prior to L-cysteine (10^−3^mol/L). A,B,C&D, representative recording of nonlabouring myometrial strip contractions. E, F&G, cumulative data of amplitude (E), frequency (F) and baseline tone (G). Data of amplitude and frequency were presented as mean (SEM) percentage of the results obtained before any drug application for each individual strip (control). For baseline muscle tone, the control was set as “0 ”. Data were presented as mean±SEM (TNL, n = 5; TL, n = 4). **P<0.01 vs control. #*P*<0.05; ##*P*<0.01 vs L-cys. L-cys: L-cysteine.

In order to confirm that the effects of L-cysteine were through producing H_2_S, CBS and CSE inhibitors were administrated before addition of L-cysteine (10^−3^ mol/L). As shown in Fig. 5, CSE inhibitor PAG (10^−4^ mol/L) partly blocked the effects of L-cysteine on the amplitude and frequency of spontaneous contractions as well as baseline muscle tone. CBS inhibitor AOAA almost blocked the effects of L-cysteine on the amplitude of spontaneous contractions, while it partly blocked L-cysteine-induced increase in baseline muscle tension and frequency of spontaneous phasic contractions. Application of PAG and AOAA at the same time abolished L-cysteine-induced decrease in spontaneous contraction amplitude and partly blocked L-cysteine-induced increase in the frequency of phasic activity and baseline muscle tension.

**Figure 6:**
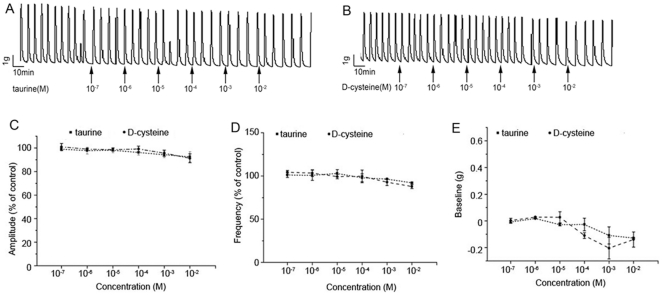
The effects of D-cysteine and taurine on myometrial contractility. Human myometrium strips were obtained from TNL patients (n = 4) undergoing elective cesarean section. Cumulative increases in D-cysteine (10^−7^–10^−2^mol/L) and taurine (10^−7^–10^−2^mol/L) were applied to myometrial strips. Spontaneous contractions and baseline muscle tension of myometrial strips were recorded. A&B, representative recording of contractions of myometrium strip treated with D-cysteine (A) or taurine (B). C,D&E, cumulative data of amplitude (C), frequency (D) and baseline tone (E). Data of amplitude and frequency were presented as mean (SEM) percentage of the results obtained before any drug application for each individual strip (control). For baseline muscle tone, the control was set as “0 ”. Data were presented as mean±SEM.

In order to ensure the specificity of L-cysteine effect on contractility of myometrium, the effects of D-cysteine and taurine on the contractility of human myometrium were tested. Neither D-cysteine nor taurine showed to affect myometrial contractility in a concentration range of 10^−7^–10^−2^ mol/L (Fig. 6).

**Figure 7:**
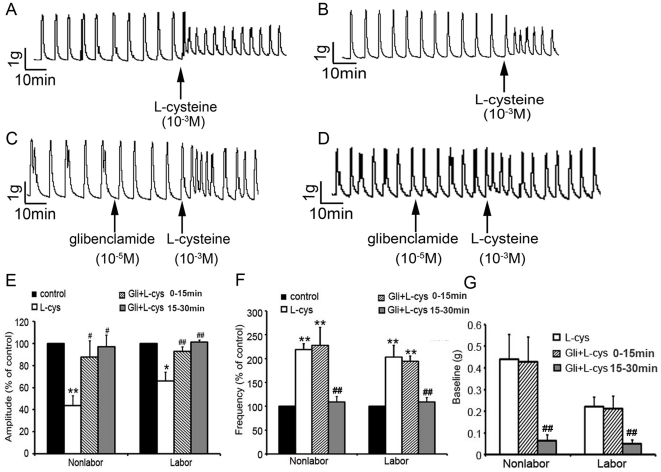
Effects of the K_ATP_ channel inhibitor on L-cysteine-induced changes in contractility of myometrium. K_ATP_ channel inhibitor glibenclamide (10^−5^mol/L) was applied to myometrial strips before addition of L-cysteine (10^−3^M). A&B, representative recording of nonlabouring(A) and labouring (B) myometrial strips were treated with L-cysteine. C&D, representative recording of nonlabouring(C) and labouring (D) myometrial strips treated with glibenclamide and L-cysteine. E,F&G, cumulative data of amplitude (E), frequency (F) and baseline tone (G). Data of amplitude and frequency were presented as mean (SEM) percentage of the results obtained before any drug application for each individual strip (control). For baseline muscle tone, the control was set as “0 ”. Data were presented as mean±SEM (TNL, n = 4; TL, n = 4). *P<0.05;**P<0.01 vs control. #*P*<0.05; ##*P*<0.01 vs L-cys. L-cys: L-cysteine.

Previous studies have demonstrated that K_ATP_ channels are involved in H_2_S modulation of smooth muscle tone[11], [22]. In the present study, the effects of L-cysteine (10^−3^ mol/L) on spontaneous contraction amplitude were abolished when the strip was pretreated with K_ATP_ channel inhibitor glibenclamide (10^−5^mol/L). Glibenclamide pretreatment did not block the stimulatory effects of L-cysteine on the frequency of spontaneous contractions and baseline muscle tone within 15 min after adminstration of L-cysteine, however, after 15 min, stimulatory effects of L-cysteine did not exist (Fig.7).

## Discussion

The present study demonstrated that CBS and CSE, the enzymes responsible for H_2_S synthesis, are present in human pregnant myometrium and mainly localized to the smooth muscle of human myometrium. The expression of enzymes as well as H_2_S production rate was significantly down-regulated during labour. Endogenous H_2_S generated by CBS and CSE modulated the spontaneous contractions and basal muscle tension of pregnant myometrium *in vitro*.

The presence of CBS and CSE has been reported in smooth muscle tissues. CSE is the main H_2_S producing enzyme in vasculature smooth muscle[11], whereas both CBS and CSE are found in gastrointestinal smooth muscle[12]. In the present study, both CBS and CSE were identified in human pregnant myometrium tissues and mainly localized to smooth muscle cells. Using the technique of real-time H_2_S measurement, it was also found that the myometrium tissues are capable of producing H_2_S after addition of the precursor L-cysteine. These results are in accordance with the study of Patel et al where they showed the presence of CBS and CSE in human uterus using Western blotting analysis[14].

The effects of endogenous H_2_S on uterine smooth muscle have not been intensively studied. Sidhu et al[21] previously showed that L-cysteine causes decreases in amplitude and frequency of spontaneous contractions of pregnant rat uterine strips *in vitro*, however, they did not show which H_2_S synthetic enzyme is involved in the action of L-cysteine. The present study demonstrated that endogenous H_2_S has dual effects on the contractility of human myomentrium as showing that L-cysteine dose-dependently inhibited spontaneous contraction amplitude in the concentration range of 10^−7^–10^−2^ mol/L, whereas it induced tonic contraction and increased the frequency of spontaneous contractions at high concentration (10^−3^mol/L). These effects could be blocked by both CSE and CBS inhibitors, suggesting that endogenous H_2_S generated by CBS and CSE can modulate the contractility of human myometrium during pregnancy.

Many studies have demonstrated that H_2_S activates K_ATP_ channel, thereby causing relaxation of smooth muscle. For instance, studies of Wang's group have suggested that vasorelaxation induced by H2S in rat aorta and mesenteric beds is mainly caused by K_ATP_ channel opening[11], [15], [23]. The study by Zhao et al has also shown that K_ATP_ channels mediate NaHS-induced inhibition of spontaneous contractions of gastric smooth muscle[24]. In consistence with these studies, our findings that glibenclamide totally abolished L-cysteine inhibition of the amplitude of phasic contractions indicates that the inhibitory effect of H_2_S on the contractility of pregnant human myometrium is mediated by K_ATP_ channels.

The dual effects of H_2_S on smooth muscle contractility have been demonstrated by some studies. Zhao et al[24]found that NaHS, a H_2_S donor, inhibits the amplitude of spontaneous contractions at high concentration (0.3–1.0mmol/L) whereas induces tonic contraction at low concentration (0.1–0.3mmol/L), which are not fully consistent with our data. Some differences in experimental conditions might be responsible for this discrepancy, for example, smooth muscle differences (‘uterine smooth muscle in our study’ vs ‘gastric smooth muscle in Zhao et al study’), reagents differences (‘ L-cysteine in our study’ vs ‘NaHS in Zhao's study’), etc. Zhao et al[24] also explored the mechanisms by which H_2_S modulates gastric smooth muscle contractility, and found that NaHS inhibition of spontaneous phasic contraction is reversed by activation of K_ATP_ channels , whereas NaHS induction of tonic contraction is prevented by voltage-gated K^+^ channel blocker. It therefore suggests that H_2_S inhibit spontaneous phasic contractions by activating K_ATP_ channels , and at meantime it can also inhibit the opening of voltage-gated K^+^ channel, causing membrane depolarization and thereby enhancing the basic tension. Lim et al (19) demonstrated that H_2_S induces vasoconstriction by downregulation of cAMP production in vascular smooth muscle cells. In the present study, as mentioned above, the inhibitory effects of L-cysteine were blocked by the K_ATP_ channel blocker glibenclamide. We also found that blockage of K_ATP_ channels reversed the excitatory effects of L-cysteine after 15 min administration of L-cysterine, but did not block the effects within 15min. Thus, the excitatory effects of H_2_S are not mediated by K_ATP_ channels. It is hard to explain the mechanisms responsible for H_2_S eliciting excitatory effects on uterine contractility at current stage. Further studies will be taken to explore the mechanisms responsible for the excitatory effects of H_2_S.

During pregnancy and labour, myometrium undergoes distinct molecular changes, thereby facilitating giving birth[1]. It has been shown that expression of various proteins including gap junction, ion channels, receptors and metabolic enzymes of uterotonic and uterorelaxant factors is up- or down-regulated in pregnant myometrium after onset of labour[1], [3]–[4], [25]. The present study also found that the expression of H_2_S synthetic enzymes in pregnant myomtrium is down-regulated during labour, and consistently, the potency of H_2_S regulating the contractility is decreased in labouring strips compared with nonlabouring strips. Although dual effects of H_2_S in the modulation of uterine contractility are found, the excitatory effect was occurred at high concentration of L-cysteine (10^−3^mol/L). The concentration of L-cysteine is about 10^−4^mol/L in peripheral circulation[26], [27]. The inhibitory effect of endogenous H_2_S generated locally is predominant *in vivo*, because the level of L-cysteine within myometrial cells is presumably lower than circulation. Thus, reduced expression of H_2_S synthetic enzymes during labour facilitates uterine contractility after onset of labour. It let us to suggests that endogenous H_2_S is one of the factors involved in transition of the relaxed pregnant uterus to the contractile state at the onset of parturition.

In conclusion, human myometrium smooth muscle cells express CBS and CSE. Endogenous H_2_S generated through CSE and CBS locally modulates the contractility of pregnant myometrium. The inhibitory effect of H_2_S on uterine contractility is through activation of K_ATP_ channels. Down-regulated expression of CBS after onset of parturition facilitates enhanced contractility of uterus during labour.

## Materials and Methods

### Ethics statement

The present study was approved by the human ethic committee of Changhai Hospital and Second Military Medical University, Shanghai, China. Informed consent was obtained from all patients. Data were analyzed anonymously.

### Tissue acquisition and preparation

Pregnant myometrial biopsies were collected at cesarean section from the following groups of pregnant women: term no labour (TNL,the average gestational age was 38 weeks, with a range of 37 to 42 weeks) and term labour (TL,the average gestational age was 38 weeks, with a range of 37 to 42 weeks). Labour was defined as regular contractions (<5 min apart) plus membrane rupture and cervical dilation (>3 cm) with no augmentation (oxytocin or PG administration). Indications for cesarean section included breech presentation, placenta previa, previous cesarean section, cephalopelvic disproportion, failure of labour to progress, fetal distress, or maternal request. The patients who had the evidence of underlying disease (e.g. hypertension, diabetes, preeclampsia, intrauterine growth restriction, etc.) were not included in this study. Myometrium tissues were taken from the upper edge of the incision line in the lower uterine segment at cesarean section. All myometrial samples were dissected free of serosa and immediately placed in Kreb's solution maintained at 4°C, and transported to the laboratory where they were stored at 4°C and used within 6 h. The Kreb's solution had the following composition: NaCl (118.0 mmol/L), KCl (4.7 mmol/l), CaCl_2_ (2.5 mmol/L), MgSO_4_ (1.0 mmol/L), KH_2_PO4 (1.0 mmol/L), glucose (11.0 mmol/L) and NaHCO_3_ (25.0 mmol/L).

### Immunohistochemistry

Human myometrial biopsies were fixed in buffered formalin prior to processing the paraffin sections. Paraffin sections (5 µm) were cut, rehydrated and microwaved in citric acid buffer to retrieve antigens. The specific antibodies for CSE and CBS were purchased from Santa Cruz Biotechnology (Santa Cruz Biotechnology, Inc. Santa Cruz, CA). Sections were incubated with 3% H_2_O_2_ to inhibit endogenous peroxidases, and then with 10% rabbit serum for 30 min to block the unspecific antibody binding. The sections were incubated with CBS or CSE antibody (1∶200) in PBS containing 1% BSA for 24h at 4°C. The bound antibodies were detected with the biotin–streptavidin–peroxidase system (UltraSensitive-SP-kit, MaiXin Biotechnology, Fuzhou, China) using diaminobenzidine (Sigma-Aldrich) as chromogen. Counterstaining was performed with hemalum. Negative controls were performed by substituting primary antibody with a normal serum in same dilution as well as primary antibody preabsorbed with a ten-fold excess of the blocking peptides.

### Total RNA extraction, RT-PCR and quantitative real-time RT-PCR

Total RNA of myometrial tissue was extracted by TRIzol reagent (Invitrogen Corp., Carlsbad, CA) following the manufacturer's instructions. 2 µg RNA was reverse transcribed using superscript reverse transcriptase (Invitrogen) and stored at −20°C.

Quantitative real-time PCR analysis was carried out in duplicates using Rotor Gene 3000 (Corbett Research, Sydney, Australia). The primers of CSE and CBS were as following: CSE (accession number NM_ 001902): 5′- GAC TCT ACA TGT CCG AAT GG -3′ and 5′-AAC CTG TAC ACT GAC GCT TCA-3′. CBS (accession number NM_000071): 5′-GGG CAC ACC ATC GAG ATC CTC-3′ and 5′-AGA GCC TGC CCA GCG TGT C-3′. Real-time PCR solution consisted of 40 ng diluted cDNA product, 0.1–0.3 µM of each paired primer, 2.5 mM Mg^2+^, 100 µM deoxynucleotide triphosphates, 2 U Taq DNA polymerase(Invitrogen) and 1 × PCR buffer. SYBR green (BMA, Rockland, ME) was used as detection dye. Quantitative real-time PCR conditions were optimized according to preliminary experiments to achieve linear relationships between initial RNA concentration and PCR product. The amplification cycles were set at 40 cycles. Amplification of the housekeeping genes β-actin and GAPDH were measured for each sample as an internal control for sample loading and normalization. The temperature range to detect the melting temperature of the PCR product was set from 60–95°C. The specificity of PCR products was examined by melting curve at the end of the amplification and subsequent sequencing. To determine the relative quantitation of gene expression for both target and housekeeping genes, the comparative Ct (threshold cycle) method with arithmetic formulae (2^−ΔΔCt^) was used[28]. Because very similar data were obtained by using either β-actin or GAPDH as an internal control, GAPDH was used for calculation of ΔCt in presentation of results.

### Western Blot Analysis

Myometrial tissues( approximately 100mg for each sample ) were homogenized in ice-cold lysis buffer consisting of 60 mM Tris-HCl, 2% sodium dodecyl sulfate (SDS), 10% sucrose, 2 mM phenylmethylsulfonyl fluoride (Merck, Darmstadt, Germany), 1 mM sodium orthovanadate (Sigma-Aldrich, St. Louis, MO), 10 µg/ml aprotinin (Bayer, Leverkusen, Germany). Then, lysates were quickly sonified in ice bath, boiled 5 min at 95°C, and stored at −80°C until used. Samples were diluted in sample buffer (250 mM Tris-HCl, pH 6.8), containing 4% SDS, 10% glycerol, 2% β-mercaptoethanol, and 0.002% bromophenol blue) and boiled for a further 5 min before loading on gel. Samples were separated on an SDS-10% polyacrylamide gel, and the proteins were electrophoretically transferred to a nitrocellulose filter at 300 mA for 90 min in a transfer buffer containing 20 mM Tris, 150 mM glycine, and 20% methanol. The filter was then blocked in TBS containing 0.1% Tween-20 (TBST) and 5% dried milk powder (wt/vol) for 2 h at room temperature. The specific antibodies, anti-CBS and anti-CSE_,_ were purchased from Santa Cruz Biotechnology (Santa Cruz Biotechnology, Inc. Santa Cruz, CA). After three washes with TBST, the nitrocellulose filters were incubated with primary antibody for CBS or CSE (1∶1000) at 4°C overnight. After another three washes with TBST, the filters were incubated with a secondary horseradish peroxidase-conjugated IgG (1∶1000) for 1 h at room temperature and further washed for 30 min with TBST. Immunoreactive proteins were visualized using the enhanced chemiluminescence Western blotting detection system (Santa Cruz). The light-emitting bands were detected with X-ray film. The resulting band intensities were quantitated using an image scanning densitometer (Furi Technology, Shanghai, China). To control sampling errors, the ratio of band intensities to β-actin was obtained to quantify the relative protein expression level.

### Real-time H_2_S production measurement

Approximately 100mg of human myometrial tissue was placed in PBS (approximately five-time volume of tissue) containing protease inhibitor (2 mM phenylmethylsulfonyl fluoride, 1 mM sodium orthovanadate and 10 µg/ml aprotinin)on ice, then tissues were diced with double scissors to small particles, washed three times with ice-cold PBS to remove red blood cells, and homogenized for 15 s[29]. The homogenate was centrifuged for 5min at 5000 rpm at 4°C to remove any remaining tissue chunks. Supernatant protein concentration was determined using a modified Bradford assay against a standard curve constructed with bovine serum albumin.

To define the real-time kinetics of H_2_S production by human myometrial tissue, a miniaturized H_2_S micro-respiration sensor (Model H_2_S-MRCh, Unisense, Aarhus, Denmark) coupled to Unisense PA2000 amplifier was used. As previously described[30]–[32], the real-time H_2_S production measurements were performed in a temperature-controlled micro-respiration chamber (Unisense) containing 1 ml of stirred (160 rpm/min) culture media (pH 7.2) at 37°C inside a well-grounded Faraday cage. H_2_S production in human myometrial homogenates was demonstrated by injecting 0.1 ml of the supernatant into the respirometer chamber. In addition, to avoid the spontaneous H_2_S oxidation, nitrogen was used to deoxygenate the culture media in the respiratory chamber before homogenates addition[33]. After the sensor signals stabilized, L-cysteine (1 mmol/L, the substrate for the enzymes CBS and CSE) and pyridoxal-5′-phosphate (PLP, 1 mmol/L, a cofactor for the enzymes CBS and CSE) were added to stimulate H_2_S production. H_2_S production rates were determined after the addition of substrate and cofactor at the initial steepest slopes of each trace. The H_2_S sensor was calibrated after each experiment with freshly prepared anoxic sodium sulfide stock solution (0–100 µmol/L) according to the manufacturer's manual, using the same buffer and conditions.

### Measurement of isometric tension in human uterine strips

The myometrium tissues were cut into about 3×3×10mm pieces, and then mounted on parallel wires and placed in a 30 ml organ bath filled with Kreb's solution maintained at 37°C ,bubbled with a gas mixture (95% O_2_–5% CO_2_). The contractile activity was measured isometrically by a tension transducer, followed by computerized recording and processing (MedLab, Nanjing, China). Each strip of myometrium was set up under an initial tension of 2 g and allowed to equilibrate for 90min, and the Kreb's solution was changed every 30 min. After equilibration, spontaneously active tissue was exposed to a cumulative increase in concentrations of L-cysteine (Sigma-Aldrich) every 30 min. In each experiment, appropriate controls were run under similar experimental conditions in strips of uterus obtained from the same woman. Only one concentration-response curve was performed in each uterine strip. In some cases, glibenclamide (Sigma-Aldrich), DL-propargylglycine(PAG)(Sigma-Aldrich) and aminooxyacetic acid hemihydrochloride (AOAA) (Sigma-Aldrich) were added to bath prior to L-cysteine.

The responses were quantified by amplitude, frequency of the contractions and baseline of muscle tone using software written specifically for this purpose. The effects were evaluated by comparing the experimental responses with the controls (set as 100%).

### Statistical analysis

The data are presented as mean±SEM. All data were tested for homogeneity of variance by Bartlett's test. The results indicated that the data were normally distributed. EC50 or IC50 value of dose response curve of L-cysteine was obtained using the software Origin 7.0. Individual comparisons were made by one-way ANOVA followed by LSD-t test. In some cases, Student's t-test was used for comparison. *P*-value <0.05 was considered to be significant.
